# ﻿A new species of *Suwallia* Ricker, 1943 (Plecoptera, Chloroperlidae) from southwestern China, with an updated key to male *Suwallia* species

**DOI:** 10.3897/zookeys.1089.72485

**Published:** 2022-03-18

**Authors:** Abdur Rehman, Qing-Bo Huo, Yu-Zhou Du

**Affiliations:** 1 School of Horticulture and Plant Protection & Institute of Applied Entomology, Yangzhou University, Yangzhou 225009, China Yangzhou University Yangzhou China; 2 Joint International Research Laboratory of Agriculture and Agri-Product Safety, the Ministry of Education, Yangzhou University, Yangzhou 225009, China Yangzhou University Yangzhou China

**Keywords:** Distribution, *Suwalliadengba* sp. nov., Tibet, Yunnan Province

## Abstract

A new species of the genus *Suwallia* Ricker, 1943 (Plecoptera, Chloroperlidae), *Suwalliadengba***sp. nov.**, is described from Tibet and Yunnan, southwestern China. A diagnosis and description of the adult habitus and aedeagal structure are illustrated with color images. Similarities in the terminalia with closely related species are discussed. In addition, an updated key to adult males of the *Suwallia* species of China is provided.

## ﻿Introduction

The family Chloroperlidae belongs to the superfamily Perloidea and is frequently referred to as “green stoneflies”. It consists of two subfamilies: Chloroperlinae Okamoto, 1912 and Paraperlinae Ricker, 1943. Presently, more than 29 species of the family Chloroperlidae are reported from China, belonging to six genera, namely: *Alloperla* Banks, 1906, *Alaskaperla* Stewart & DeWalt, 1991, *Haploperla* Navás, 1934, *Suwallia* Ricker, 1943, *Sweltsa* Ricker, 1943 and *Utaperla* Ricker, 1952 (Wu 1938; Nelson and Hanson 1968; Du 1999; Li and Wang 2011; Li et al. 2013, 2014, 2015a, b; Chen and Du 2015, 2016a, b, 2017; Dong et al. 2018; Yang and Li 2018; Chen 2019; Mo et al. 2020; Shi et al. 2022).

The genus *Suwallia* Ricker, 1943 belongs to tribe Suwalliini Surdick, 1985 of the subfamily Chloroperlinae. It is distributed in the East Palearctic and Nearctic regions (DeWalt et al. 2021). Most species of the genus *Suwallia* were revised and recorded by Alexander and Stewart (1999). *Suwallia* is mainly distributed in Russia, Mongolia, Japan, and North America (Alexander and Stewart 1999; Teslenko and Zhiltzova 2009; Judson and Nelson 2012). In China, the first species of *Suwallia* was reported by Li et al. (2015a), and until now seven species of this genus had been reported for the country: *Suwalliaerrata* Li & Li, 2021, *Suwalliadecolorata* Zhiltzova & Levanidova, 1978, and *Suwalliatalalajensis* Zhiltzova, 1976 were reported by Li et al. (2015a, b) and Li et al. (2021) from the Inner Mongolia Autonomous Region, northern China (Fig. 7), whereas *Suwalliawolongshana* Du & Chen, 2015 and *Suwalliajihuae* Chen, 2019 were reported by Chen and Du (2015) and Chen (2019) from the Sichuan Province of southwestern China. Recently, *Suwalliakuandian* Shi, Wang & Li, 2022 and *Suwalliaasiatica* Zhiltzova & Levanidova, 1978 were reported by Shi et al. (2022) from Liaoning Province, northeastern China. In the current paper, a new species of *Suwallia* is described from Tibet and the Yunnan Province of southwestern China. This is the first record of the *Suwallia* genus from both regions. Tibet is also known as Xizang in Chinese and is positioned on the Tibetan plateau, known as the world’s highest and largest plateau. The Yunnan Province lies adjacent to the Tibet, Sichuan, Guizhou, and Guangxi provinces of China and borders with Myanmar, Laos, and Vietnam. The taxonomy of the new species is discussed, a distributional map, and a key to the known species of *Suwallia* from China are provided.

## ﻿Materials and methods

All specimens were collected by aerial net or hands and preserved in 75% ethanol. Terminalia were examined and illustrated by KEYENCE VHX-5000 and the final images were prepared using Adobe Photoshop CS6. The type specimens of the new species were placed in the insect collection of Yangzhou University **(ICYZU)**, Jiangsu Province, China. Data for the key and distribution map were extracted from the published literature (Chen and Du 2015; Li et al. 2015a, b; Chen 2019; Shi et al. 2022).

## ﻿Results

### ﻿ Suwallia dengba sp. nov.

Taxon classificationAnimaliaPlecopteraChloroperlidae

73CEA26E-B2AB-5739-8B9B-A3BB407A9C56

http://zoobank.org/51F6012D-7AB2-4F16-9095-2B1B9E7CE5BE

Figs 1 , 2 , 3 , 4 , 5 , 6 , 7 , 8

#### Type material.

***Holotype***, 1♂, China, Tibet Autonomous Region, Dengba village, Mangkam County, Qamdo city, 3437 m, 29°32.406'N, 98°13.425'E, 18.IX.2019, Leg. Huo Qing-Bo (ICYZU). ***Paratypes***, 6♂♂, 6♀♀, data same as holotype (Figs 7, 8); 5♂♂, 17♀♀, Yunnan Province, Diqing Tibetan Autonomous Prefecture, Shangri-la city, on the way from Diqing to Gezan Township, 3445 m, 27°45.656'N, 99°56.374'E, 7.IX.2019. Leg. Huo Qing-Bo (ICYZU); 2♂♂, 4♀♀, China, Yunnan Province, Diqing Tibetan Autonomous Prefecture, on national highway (G214) near Tongduishui and Deiyong Benglao, 3432 m, 28°18.282'N, 99°8.472'E, 9.IX.2019, Leg. Huo Qing-Bo (ICYZU); 1♂, 2♀, China, Yunnan Province, Diqing Tibetan Autonomous Prefecture, on national highway (G214) near Zhubagong, Deqin County (Fig. 7), 4027 m, 28°23.885'N, 98°59.143'E, 10.IX.2019, Leg. Huo Qing-Bo (ICYZU).

#### Diagnosis.

The new species is characterized by the sclerotized median sclerite of tergum X and its aedeagus armature. The shape of the median sclerite of tergum X resembles a turtle or a hexagonal star. The aedeagus, with a large distinct sclerite divided into an eagle-shaped trifurcate structure, the large median sclerite, and one pair of wing-shaped lateral sclerites on both sides, is diagnostic (Figs 2–4).

#### Description.

**Figure 1.**
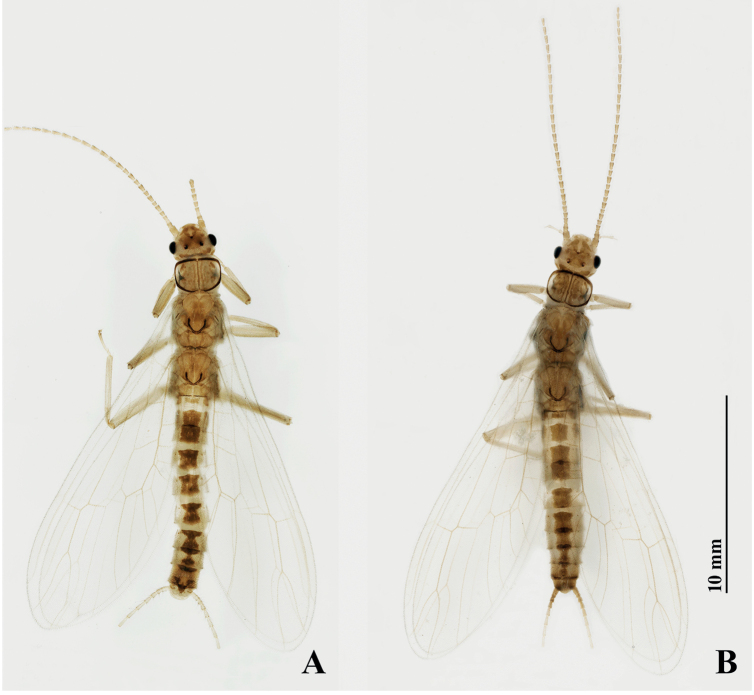
*Suwalliadengba* sp. nov. **A** male habitus **B** female habitus.

**Figure 2.**
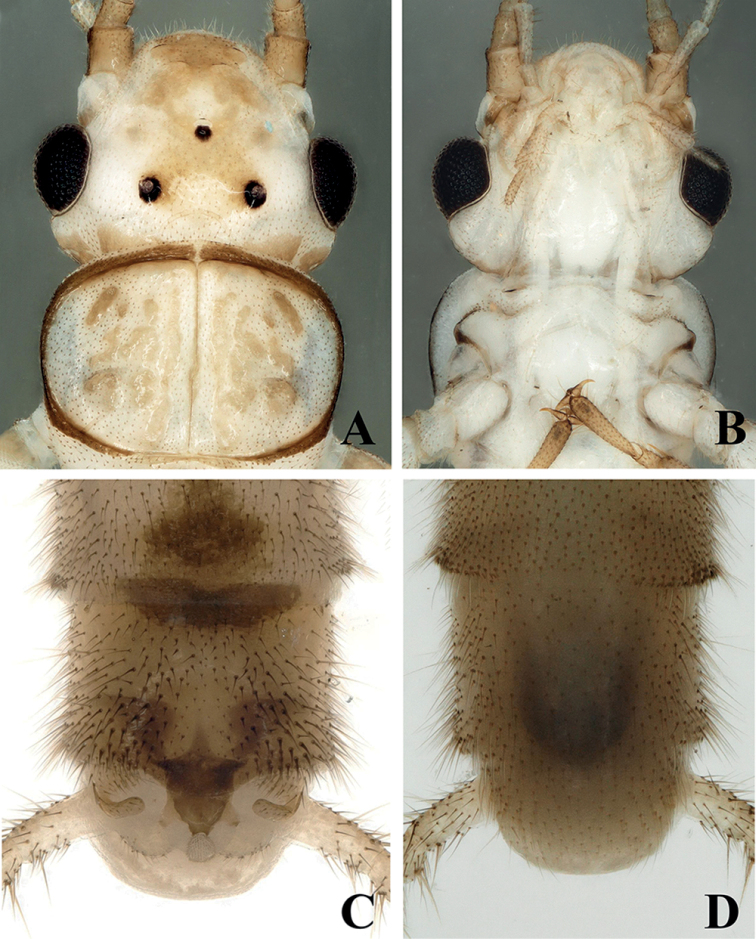
*Suwalliadengba* sp. nov. Holotype male **A** head and prothorax, dorsal view **B** head and prothorax, ventral view **C** terminalia, dorsal view **D** terminalia, ventral view.

***Adult habitus*** (Fig. 1A). Adult body length 8.5–9.5 mm (N = 10), forewing length 6.5–7.5 mm, hindwing length 5.5–6.5 mm. General color of body pale yellow in alcohol. Triocellate, head yellowish-white to yellowish-brown. Ocellar triangle and frontoclypeal area pale yellowish-brown, antenna pale brown, covered with small brown to dark brown setae. Pronotum disc margins covered with dark brown bands and with a thin dark medial stripe (Fig. 2A). Legs pale brown, mesonotum and metanotum with a distinct dark brown U-shaped marking, wings hyaline with yellow venation. Abdominal terga I–VIII with a wide medial trapezoidal dark brown stripe, slightly constricted medially on terga VII and VIII (Figs 1A, 2C–D).

**Figure 3.**
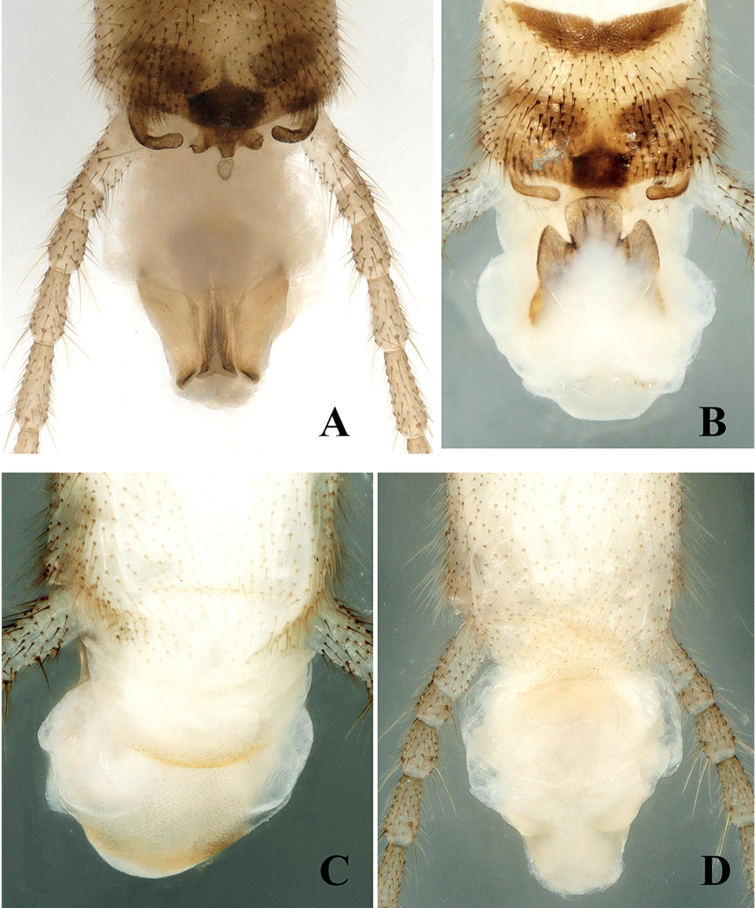
*Suwalliadengba* sp. nov. Male paratype. **A** terminalia with aedeagus, dorsal view **B** aedeagus everted, dorsal view **C** aedeagus, caudal ventral view **D** aedeagus, ventral view.

**Figure 4.**
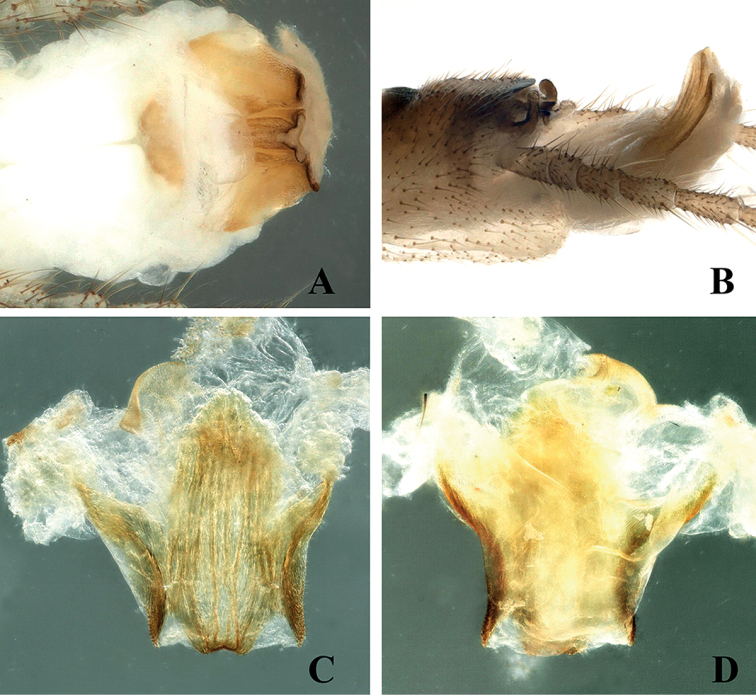
*Suwalliadengba* sp. nov. **A** aedeagus **B** terminalia, lateral view **C** aedeagal sclerite, dorsal view **D** aedeagal sclerite, ventral view.

**Male** (Figs 2–4). Tergum IX concave medially with semicircular stripe anteriorly, posteriorly covered with dark brown, thick hairs. Tergum X divided, median portion with a distinct dark brown sclerite resembling a turtle or hexagonal star in dorsal view (Figs 2C, 6A). Hemitergal processes sclerotized, with tiny hairs, finger-shaped and curved forward. Epiproct membranous, circular, knob-like, covered with minute hairs. Sternum IX ventrally extended anteriorly (Fig. 2D). Aedeagus membranous with a distinct sclerotized sclerite after eversion. Aedeagal sclerite resembling an eagle, divided into a trifurcate structure, a large median sclerite, and one pair of lateral sclerites (Figs 3A, 4A–D, 6B). Lateral sclerites armed with minute scales. Membranous part of aedeagus with fine cuticular asperities (Fig. 3A–D).

**Figure 5.**
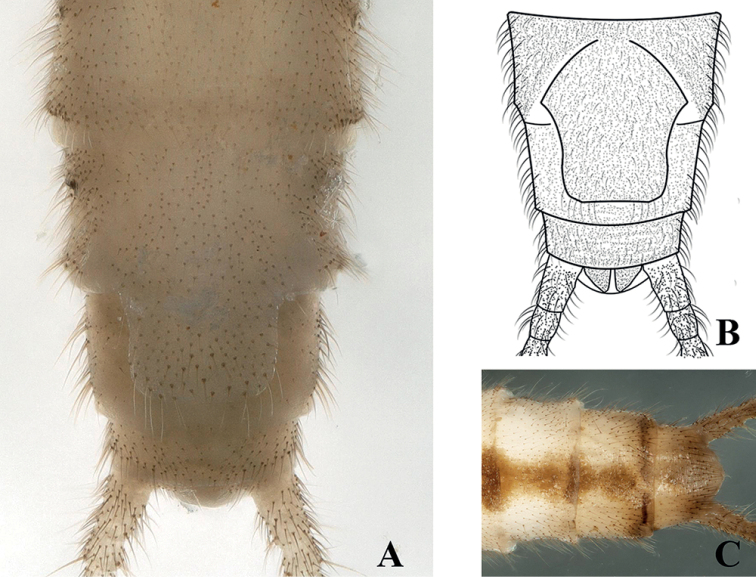
*Suwalliadengba* sp. nov. Female paratype. **A** terminalia, ventral view **B** terminalia, ventral view **C** terminalia, dorsal view.

**Female. *Adult habitus*** (Fig. 1B). Body length 9.0–10 mm (N = 10), forewing length 7.5–8.5 mm, hindwing length 6.5–7.5 mm. General body color, shape and appearance similar to those of male. Head and pronotum similar. Dorsal segment of abdomen with trapezoidal dark brown stripe extended to sternum VIII, subgenital plate large, extending to posterior portion of sternum IX, constricted from base, expanded medially, then slightly tapering toward posterior margins. Subgenital plate covered with minute, fine hairs. Tergum X not produced posteriorly. Paraproct in the shape of a small triangle, bearing small hairs (Fig. 5A–C).

#### Egg and nymph.

Unknown.

#### Distribution.

Southwestern China (Tibet and Yunnan Province).

#### Etymology.

The species is named after the type locality, Dengba village.

#### Remarks.

**Figure 6.**
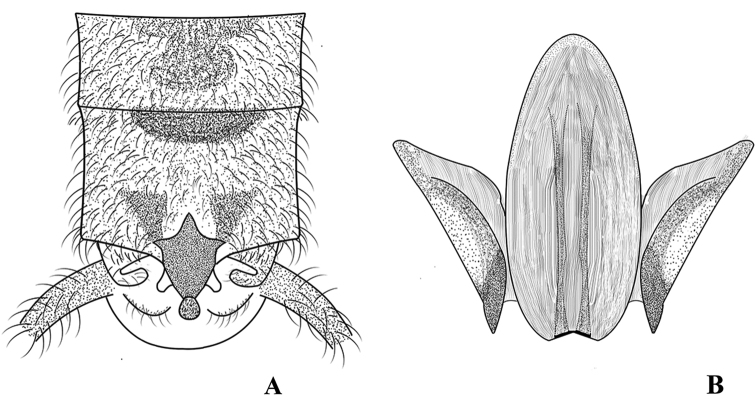
*Suwalliadengba* sp. nov. **A** male terminalia, dorsal view **B** aedeagal sclerite.

The new species is closely related to *Suwalliatalalajensis*, but can be distinguished by the sclerotized portion between the hemitergal processes, the pigmentation of tergum IX, the armature of the aedeagus and the well-developed, membranous, knob-like epiproct. *Suwalliatalalajensis* does not have a distinct aedeagal sclerite (Li et al. 2015b: fig. 5), whereas the new species has a distinct sclerite (Figs 4A–D, 6B). Tergum IX of the new species is covered with abundant, thick hairs, and its body pigmentation is different from that of *Suwalliatalalajensis*. The new species also shows similar characteristics to *Suwalliaerrata* (Li et al. 2021), but it can be easily differentiated by the sclerotized portion between the hemitergal process and the shape of the aedeagus. *Suwalliaerrata* has a V-shaped aedeagal sclerite (Li et al. 2015a: figs 1–6), but the new species has the aedeagal sclerite of a different shape. The new species lives in fast-flowing rivers (width = 5 m), where a large gravel substrate is present. The adults occur on leaves of trees or shrubs near the river (Fig. 8).

**Figure 7.**
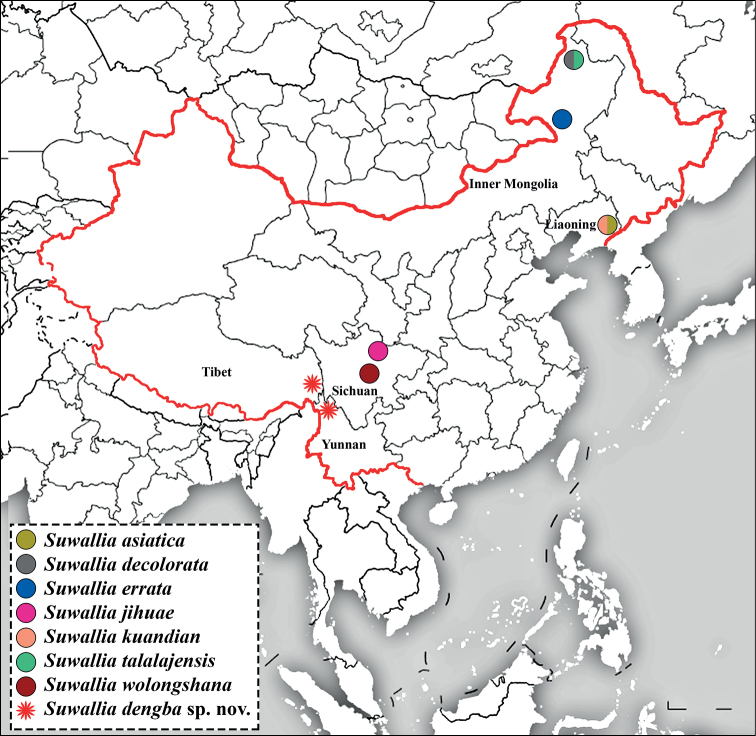
Revised map showing distribution of *Suwallia* species in China (modified from www.tianditu.gov.cn).

**Figure 8.**
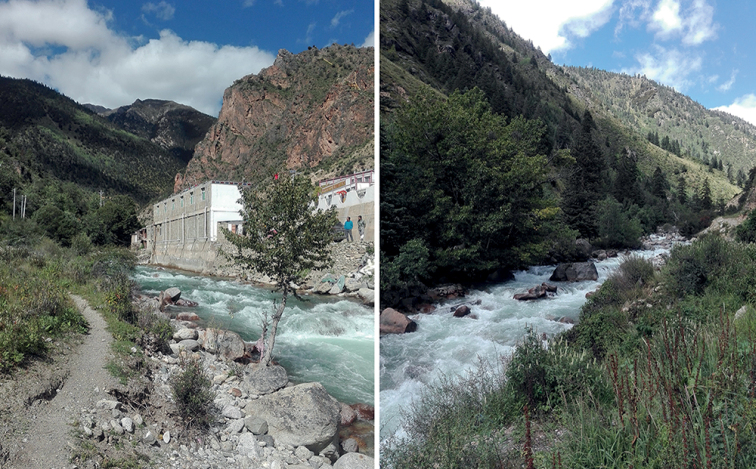
Habitat at the type locality of *Suwalliadengba* sp. nov. Specimens were collected from the small trees and grasses near the stream (photograph Huo Qing-Bo).

### ﻿Key to adult males of *Suwallia* species from China (modified from Chen 2019)

**Table:** 

1	Epiproct reduced, tergum X with two median sclerites	**2**
–	Epiproct well developed, tergum X with undivided median sclerite	**3**
2	Tergum X with two longitudinal median sclerites (see Chen and Du 2015: figs 1–8)	***Suwalliawolongshana***
–	Tergum X with H-shaped median sclerite (see Chen 2019: fig. 3)	***Suwalliajihuae***
3	Tergum X with V-shaped median sclerite, aedeagus membranous, without spines or structures (see Shi et al. 2022: fig. 2)	***Suwalliaasiatica***
–	Tergum X median sclerite triangular or subrectangular in shape, aedeagus with spines or structures	**4**
4	Tergum X median sclerite triangular in shape, epiproct small, aedeagus with triangular spines forming T-shaped structure (see Li et al. 2015b: fig. 2)	***Suwalliadecolorata***
–	Tergum X median sclerite not as above, epiproct well developed and knob-like	**5**
5	Tergum X medial sclerite subrectangular, anterior margins with two separate sclerites	**6**
–	Tergum X median sclerite of turtle or hexagonal shape	**7**
6	Tergum X anterior margins divided into two sclerites, epiproct with long hairs and without posterolateral bifurcation, aedeagus with V-shaped sclerite (see Li et al. 2015a: figs 1–6)	***Suwalliaerrata***
–	Tergum X anterior margins with two separate paramedial sclerites, arch-shaped in lateral view, epiproct with stout posterolateral bifurcation, aedeagus with triangular sclerite, lateral margins darker (see Shi et al. 2022: fig. 1)	***Suwalliakuandian***
7	Tergum X median sclerite turtle-like, aedeagus membranous, without distinct armature or sclerite (see Li et al. 2015b: fig. 2)	***Suwalliatalalajensis***
–	Tergum X median sclerite hexagonal star-shaped, pointed posteriorly, aedeagus with distinct trifurcate sclerite (Figs 2–4)	***Suwalliadengba* sp. nov.**

## Supplementary Material

XML Treatment for Suwallia dengba
